# Supplementary material for: CHRAC/ACF contribute to the repressive ground state of chromatin
Source: Life Sci Alliance. 2018 Feb 9;1(1):e201800024. doi: 10.26508/lsa.201800024 (PMC6238394; doi:10.26508/lsa.201800024)
Supplement: Supplementary file 3 [file LSA-2018-00024_TableS3.docx]

| **Primer** | **Sequence** | **Usage** |
| --- | --- | --- |
| Acf_Rec_NF | GCCATTTAAACTTAAGGACATTCAAGAGCAAAAGGAAAACACCAAACATGGAAGTGCATACCAATCAGGAC | recombineering |
| Acf_Rec_NR | TTCTTGCCCTCCTTCTGATTCAGGTCGAATCCTTCCCGCTTGCAAATGGGCTTGTCGTCGTCATCCTTGTA | recombineering |
| Acf_Rec_CF | TGCAGCTACCGTTTAGGCCTAGCGATATGAACGGGGAAGTCAAAGCTTGCGAAGTGCATACCAATCAGGAC | recombineering |
| Acf_Rec_CR | GTAGACTAACTAATTTACACGATAGCTGGTGGAGATCAGCGTCCGGCTCACTTGTCGTCGTCATCCTTGTA | recombineering |
| UAS_ChIP_f1 | TCCGAGCGGAGACTCTAGC | ChIP-qPCR, UAS |
| UAS_ChIP_r1 | TTTGCTTGTTTGAATTGAATTGTC | ChIP-qPCR, UAS |
| UAS_ChIP_f2 | TAGGGAATTGGGAATTCGAG | ChIP-qPCR, 5' of LacZ |
| UAS_ChIP_r2 | TTAGCAGGCTCTTTCGATCC | ChIP-qPCR, 5' of LacZ |
| Spt4_fw | GCTCCGATTCATAAGCCCAG | ChIP-qPCR, negative control region |
| Spt4_rv | GCCTCTTTCGGAGCAGCTTT | ChIP-qPCR, negative control region |
| LacZ_RT_f1 | CGCTAGAGTCGACCAATTCC | RT-qPCR |
| LacZ_RT_r1 | GGCAACGAAAATCACGTTCT | RT-qPCR |
| RpII-140_fw | GCGCTATGGGTAAGCAAGCT | RT-qPCR, control |
| RpII-140_rv | TCACAAGTGGCTTCATCGGA | RT-qPCR, control |
| Acf1_crispr_LHS6 | TTAGCGCAGCGTCCACCTAA | CRISPR/Cas9, gRNA |
| Acf1_crispr_RHS2 | GGGCGCCTACCAGCTAAACG | CRISPR/Cas9, gRNA |

**Supplementary Table 3**
